# Evidence of zoonotic rickettsiae in ixodid ticks of domestic animals in some communal farms in the Eastern Cape Province, South Africa

**DOI:** 10.5455/javar.2024.k771

**Published:** 2024-06-04

**Authors:** Olusesan Adeyemi Adelabu, Benson Chuks Iweriebor, Chikwelu Larry Obi

**Affiliations:** 1 SAMRC Microbial Water Quality Monitoring Centre, University of Fort Hare, Alice, South Africa; 2 School of Science and Technology, Sefako Makgatho Health Sciences University, Ga-Rankuwa, South Africa

**Keywords:** *Rickettsia*, *Borrelia*, zoonosis, emerging, domestic animals, South Africa

## Abstract

**Objective:**

The abundance of tick populations in South Africa represents a probable risk for both animal and human health. *Rickettsia* spp. and *Borrelia* spp. are well-known agents of emerging human tick-borne infectious diseases worldwide. Nevertheless, the epidemiology of their infections has been underreported in South Africa. Therefore, this study aimed to profile zoonotic *Rickettsia* and *Borrelia* species from ticks infesting domesticated animals in the Eastern Cape, South Africa.

**Materials and Methods:**

Morphological and molecular identification techniques were conducted on 1,200 tick samples collected from domestic animals before screening for the target bacterial pathogens. The molecular identification of the tick samples was based on the amplification of the 12S rRNA mitochondrial Deoxyribonucleic acid. At the same time, those of *Rickettsia* and *Borrelia* species were carried out by amplifying fragments of *glt*A and *omp*B genes for *Rickettsia* and *fla*B gene for *Borrelia *spp. Thereafter, the positive amplicons for *Rickettsia omp*B were sequenced and further analyzed. Borrelia PCRs were negative; therefore, sequencing could not be performed.

**Results:**

Eight species of ticks belonging to three genera; *Rhipicephalus, Amblyomma, *and *Haemaphysalis, *were identified. A total of 27% (320/1,200) samples were confirmed positive for *Rickettsia,* of which 23% (74/320) were positive for *omp*B genes. Phylogenetic analysis of *omp*B revealed a high homology to rickettsial reference strains from GenBank, with no positive result for *Borrelia. *The generated sequences showed homology with *R. africae*-KX227790 (100%), *R. parkeri*-KY113111 (99.8%), *R. peacockii *(99.3%), and* R. slovaca*-JX683122 (99.1%) representative sequences in GenBank.

**Conclusion:**

The findings from this study revealed that ticks harbored *Rickettsia* species with possible zoonotic potential.

## Introduction

Vector-borne diseases constitute a severe risk to human health, causing substantial morbidity and mortality worldwide [1]. Ticks are hematophagous ectoparasites of vertebrates that obtain their nutrition by feeding on blood; hence, they have been described as competent vectors of diseases. Over 10% of the currently known species diversity has been described to be of medical or veterinary significance [2].

After mosquitoes, ticks are considered the second most important vector of human diseases and the primary vector of pathogenic organisms in animals [3], as well as the most important vector for numerous severe zoonotic infections worldwide [4]. Also, an increase in the range of tick-borne diseases (TBDs) infecting domestic animals and humans has been observed recently, and several significant zoonotic TBDs such as rickettsioses [5] and Lyme borreliosis [6] are on the increase worldwide. Tick-borne pathogens (TBPs) have been reported to maintain lifecycles that include ticks and animals, and sometimes they are transmitted to humans, who are usually the dead-end hosts [6].

*Rickettsia* and *Borrelia* spp. are both transmitted by ticks and body lice. They are among the numerous zoonotic pathogens responsible for febrile illness among humans [6]. Rickettsial diseases, caused by organisms of the genus *Rickettsia* are classified into three bio-groups; the first bio-group is known as the spotted fever group (SFG), which includes rocky mountain spotted fever caused by *R. rickettsii, as well as other spotted fevers such as Boutonneuse fever (Mediterranean spotted fever, Kenya tick-bite fever, Israeli spotted fever, African tick typhus, Marseilles fever, and Indian tick typhus,) caused by several other Rickettsia species. Second is the typhus group (TG) rickettsiae,* which are responsible for similar diseases but with different epidemiology [7], and the etiologic agents are *R. typhi *and *R. prowazekii*. However, they have been described to be similar to causative agents for the SFG but are distinct antigenically. Finally, a translational group includes *Rickettsia felis*, *Rickettsia australis*, and *Rickettsia akari*. Rickettsial diseases have been reported to be very challenging to diagnose, owing to similar symptoms and epidemiology shared with several other febrile illnesses. Thus, suggesting that the overall reported cases of rickettsial diseases are probably inaccurate as they are often underreported [8].

*Borrelia*, a genus of bacteria belonging to the *Spirochaetaceae* phylum [9], is a causative agent of borreliosis and a zoonotic infectious disease transmitted by ticks. Over 50 species of *Borrelia* have been categorized into two groups. The first group comprises about 21 species transmitted by the hard ticks within *Borrelia burgdorferi *sensu lato complex and is related to the Lyme borreliosis group, while 19 species are described to be mainly transmitted by soft ticks associated with relapsing fever group except the human louse-borne *Borrelia recurrentis* [10].* Borrelia* species exist in enzootic cycles mostly involving ticks and several animals and bird hosts.

Owing to the expansion in geographical boundaries by ticks into new ecological terrains, TBPs belonging to the *Rickettsia* and *Borrelia* genera that were previously considered to be endemic to a particular geographical location are now being discovered in different ticks from different parts of the world [11]. The understanding of bacteria transmitted by ticks (potential reservoirs and vectors of microorganisms) in a given geographical location is a valuable marker for assessing the risk of infection in both humans and animals. The Eastern Cape Province of South Africa is largely a rural settlement, with animal husbandry being one of the major occupations for most households. Also, the Province has game reserves, which are sites of attraction for tourists. Cattle and other domesticated animals are kept in close proximity to homes and are allowed to graze freely in the vegetation close to game reserves where they are in close contact with the wild, which presents an avenue for direct/indirect contact with infected ticks from the wild, thus making the spread of zoonotic pathogens possible to the public. *TBDs* in humans have been reported in South Africa [11]. This study, therefore, was aimed at investigating the prevalence of TBPs of *Rickettsia* and *Borrelia* spp. in ticks parasitizing domesticated animals in the Amatole and O.R Tambo District Municipalities of Eastern Cape, South Africa.

## Materials and Methods

### Ethical clearance

Ethical approval and clearance certificate (REC-270710-028-RA) were obtained from the University of Fort Hare Research and Ethics Committee (UREC). Permission to collect samples was sought from the farmers and verbal consent was given by the cattle owners and herdsmen (Mr. Kumalo Temba, Ryan Sbusiso, and Gerald Michael).

### Sample collection

**Figure 1.**
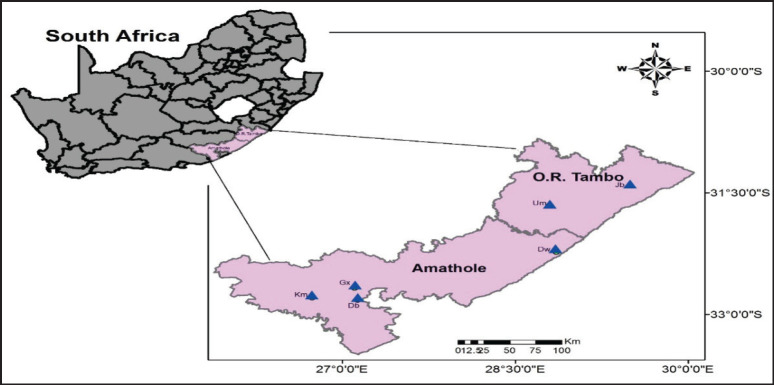
The map showing the geographical location of the sampling sites with their coordinates; Debe (Db) = 32°52‘11.852“S, 27°1‘14.171“E; Gxulu (Gx) = 32°40‘26.702“S, 27°6‘19.591“E; KwaMemela (Km) = 32°47‘38.497“S, 26°44‘10.889“E; Dwesa (Dw) = 32°13‘50.916“S, 28°51‘16.135“E; Umtata (Um) = 31°39‘26.69“S, 28°48‘0.194“E; Jambini (Jb) = 31°23‘36.856“S, 29°29‘46.921“E. Map created using ArcMap 10.5.1.

Between July 2017 and April 2018, adult ticks were manually removed from farm animals into sterile 50 ml Nalgene tubes containing 70% ethanol, with the assistance of animal health technicians and animal farmworkers. The six different sampling sites selected for this study are known geographical locations for animal husbandry in Amatole and O.R Tambo District Municipalities of the Eastern Cape, South Africa (Fig. 1). There was adherence to the University of Fort Hare Animal Ethics Committee regulations on animal handling throughout the sampling period. The collected ticks were transported to the Applied and Environmental Microbiology Research Group laboratory in the Department of Biochemistry and Microbiology at the University of Fort Hare for analyses. Collected ticks from different animals and locations were properly labeled in different tubes for easy identification and to avoid possible mix up.

### Tick identification and deoxyribonucleic acid (DNA) extraction

Upon arrival at the laboratory, identification of tick species was carried out based on morphologic criteria such as scutum formation, capitulum formation, and limb formation [12]. Upon identification, the ticks were washed in sterile distilled water about 3 to 4 times for total removal of ethanol. The engorged ticks were chopped individually while the non-engorged ticks were pooled (4 per pool according to tick species) together and chopped with a sterile blade in a petri dish containing phosphate buffer saline, then transferred into a 2 ml centrifuge tube and vortexed. Following this process, DNA extraction was carried out using the commercially available kit, Promega ReliaPrep^®;^ gDNA Tissue Miniprep System (Madison, USA), and the manufacturer’s protocol was strictly adhered to. Each tick sample was processed using the method previously described by Madison-Antenucci et al. [13].

### Molecular identification of tick species

For the molecular identification of tick species, a fragment of mitochondrial 12S ribosomal RNA (rRNA) gene was amplified using a set of oligonucleotides (85F 12S F:5‘-TTA AGC TTT TCA GAG GAA TTT GCT C-3‘ and 2225 12S R:5‘-TTT AAG CTG CAC CTT GAC TTA A-3‘), as previously described by Pesquera et al. [14]. The polymerase chain reaction was performed in a 25 µl reaction mixture comprising 14 μl of master mix (GoTaq^®;^ G2 Green Mastermix), 1 μl each of 10 pmol/l of the forward and reverse primers, 4 μl of RNase nuclease-free water, and 5 μl of DNA template. The cycling conditions used for the amplification were as follows; initial denaturation at 94°C for 3 min, followed by denaturation at 93°C for 30 sec, annealing at 51°C for 30 sec, and elongation at 72°C for 60 sec with a final elongation at 72°C for 5 min.

### Molecular detection of tick-borne bacterial pathogen

#### Rickettsia species

For the detection of rickettsiae from the extracted DNA through polymerase chain reaction (PCR), a fragment of *glt*A gene was amplified using the following genus-specific primer pair (*glt*A: F:5¢-TTT GTA GCT CTT CTC ATC CTA TGG C-3’ and *glt*A: R:5¢-CCC AAG TTC CTT TAA TAC TTC TTT GC-3¢) as previously described by Iweriebor et al. [15]. The reaction mixture containing 25 μl volume consisted of 14 μl of master mix (GoTaq^®;^ G2 Green Mastermix), 1 μl each of 10 pmol/l of the forward and reverse primers, 4 μl of RNase nuclease-free water, and 5 μl of DNA template. DNA amplification was carried out using the Biorad T100^®;^ thermal cycler system, with the following cycling conditions; initial denaturation at 94°C for 3 min, followed by denaturation at 93°C for 30 sec, annealing at 48°C for 30 sec, elongation at 72°C for 60 sec with a final elongation at 72°C for 5 min. Positive control of *Rickettsia* species (KX891173) was added to the reaction. Subsequently, all the positive samples were further subjected to screening for outer membrane proteins B (*omp*B) using the primer pair *omp*B: F: 5¢-GTA ACC GGA AGT AAT CGT TTC GTA A-3¢ and *omp*B: R:5¢-GCT TTA TAA CCA GCT AAA CCA CC-3’ in a PCR mixture and cycling conditions as previously described by Pesquera et al. [14], with slight modification of the annealing temperature. Negative control was included in the PCR so as to detect false positives or any possibility of cross-contamination.

### *Borrelia* species

Two sets of primers were used to amplify a partial region of *fla*B gene for *Borrelia* species: outward and inward primers (A two set of primers were used to amplify a partial region of flab gene for *Borrelia* species; outward primer pairs of *flab*: F: 5’-CCG TGC TAA TTG TAG GGC TAA TAC-3’ and flab: R: 5’-GAA GGT GCT GTA GCA GGT GCT GGC TGT-3’] while the inward primers of *flab*: F: 5’-AAR GAA TTG GCA GTT CAA TC-3’ and *flab*: R: 5’-GCA TTT TCA ATT TTA GCA AGT GAT G-3’). Nested PCR mixtures and cycling conditions were as previously described by Cherry et al. [16]. All amplified PCR products were visualized via transillumination on 1.5% agarose gel stained with ethidium bromide. Negative control was included in the PCR to detect false positives or any possibility of cross-contamination.

Bi-directional sequencing was carried out on all the positive *omp*B amplicons and on the 12S rDNA amplicons for tick identification, using ABI3,500xl automated DNA sequencer with a 50 cm Capillary array and POP7 (all supplied by Applied Biosystems).

### Sequencing, BLAST, and phylogeny analyses

Nucleotide sequences for both forward and reversed strands were assembled and edited to generate consensus sequences for each positive PCR product, using the Geneious program. version 10.1.2.

The consensus sequences data generated after editing were subjected to the BLAST program in GenBank for homology search with other curated sequences (http://blast.ncbi.nlm.nih.gov). The search parameters were set on highly similar sequences; hence, *Rickettsia* spp. was chosen separately as the organism option. Sequences with a percentage similarity above 97% were downloaded for phylogenetic analysis.

For *Rickettsia* species, 320 (27%) were positive *glt*A genes, out of which 74 (23%) were further confirmed positive for *omp*B gene. In contrast, no positive sample was detected for *Borrelia*. A homology search for the generated sequences from this study revealed a high percentage of similarity between 98% and 100% with other homologous *omp*B of different *Rickettsia* sequences in GenBank (Table 2).

**Figure 2.**
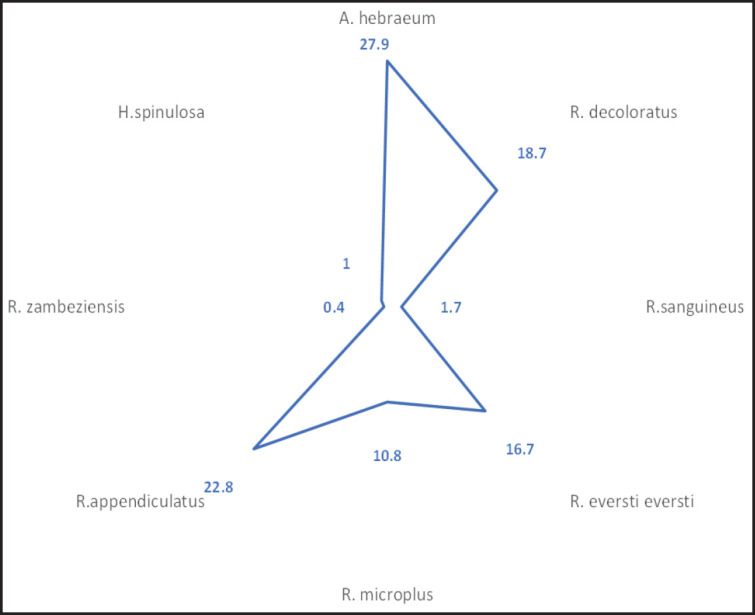
Prevalence of tick species collected in the study. Figure shows the overall prevalence of tick species collected in all the sampling sites.

The derived *Rickettsia* (*omp*B) sequences were further subjected to phylogenetic analyses with the selected *Rickettsia omp*B reference strains from GenBank. The reference sequences were previously aligned with the derived sequences, using ClustalW in MEGA 7.0. version software before generating the phylogenetic tree as shown in Figure 2.

## Results

A total of 1,200 ticks were manually removed from domesticated ruminants (718, 130, and 352 from cattle, sheep, and goats, respectively) from selected communal farms from Amatole and O.R Tambo District Municipalities. Eight species of ticks belonging to three genera; *Rhipicephalus, Amblyomma, *and* Haemaphysalis, *were identified in this study (Table 1 and Fig. 2), with *Amblyomma hebraeum *having the highest occurrence of 335 (27.9%), followed by *R. appendiculatus*; 274 (22.8%), *Rhipicephalus decoloratus*; 224 (18.7%) and *Rhipicephalus eversti eversti*; 200 (16.7%). For *Rickettsia* species, 320 (27%) genetic materials (DNA) were positive for *glt*A genes, out of which 74 (23%) were further confirmed positive for *omp*B gene while none was positive for *Borrelia* spp. *flab*. A homology search for the generated sequences revealed a high identity with other homologous *omp*B of other *Rickettsia* sequences in GenBank (Table 2).

### Phylogenetic analysis of tick species

**Figure 3.**
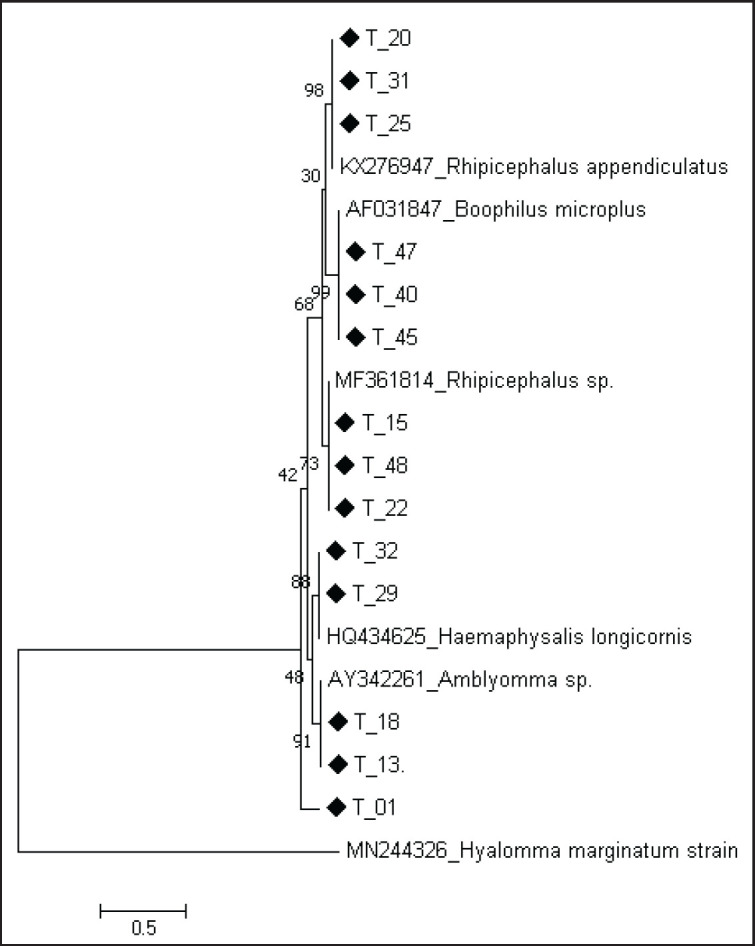
Genetic relatedness of tick species from the study and reference strains from GenBank using nucleotide sequences of mitochondrial 12S ribosomal RNA gene. Tick sequences obtained in this study are marked with square dots.

Phylogenetic analysis of generated tick sequences showed that the three genera; *Rhipicephalus, Amblyomma, *and* Haemaphysalis* were initially identified through morphologic criteria, clustered with different corresponding species of the reference sequences (Fig. 3). Sequence T01, T13, and T18 were shown to cluster with reference sequences AY342261-*Amblyomma*. sp. Sequence T29 and T32 were shown to cluster closely with reference strain HQ434625-*H. longicornis*. Likewise, sequences T20, T25, T31, clustered with reference strain KX276947-*R. appendiculatus* and *Rhipicephalus* sp. Finally, sequences T40, T45, and T47 were found to cluster with reference sequence AF031847-Rhipicephalus microplus*. *The evolutionary history was inferred using the Maximum Likelihood method. Initial tree(s) for the heuristic search were obtained automatically by applying Neighbor-Join and BioNJ algorithms to a matrix of pairwise distances estimated using the maximum composite likelihood (MCL) approach and then selecting the topology with superior log likelihood value. The bootstrap consensus tree inferred from 1,000 replicates was taken to represent the evolutionary history of the taxa analyzed. Evolutionary analyses were conducted in MEGA7.

**Table 1.** Diversity of tick species and number of positive *Rickettsia* samples.

Tick species	Number of tick species per animal			Total number of ticks (%)	Numbers of positive samples for *Rickettsia* spp. (%)
	Cattle	Goat	Sheep		
*A. hebraeum*	235	80	20	335 (27.9)	140 (42.0)
*R. decoloratus*	129	70	25	224 (18.7)	
*R. sanguineus*	0	15	5	20 (1.7)	
*R. eversti eversti*	140	40	20	200 (16.7)	78 (39.0)
*R. microplus*	70	40	20	130 (10.8)	
*R.appendiculatus*	139	95	40	274 (22.8)	102 (37.2)
*R. zambeziensis *	5	0	0	5 (0.4)	
*H.spinulosa*	0	12	0	12 (1.0)	
Total	718	352	130	1,200	

*R = Rhipicephalus, A = Amblyomma, H = Haemaphysalis.

**Table 2.** Similarities of rickettsial (*omp*B) sequences obtained from tick samples.

Sample	Blast homology (%)	Reference species	GenBank accession
D189	100.0	*R. africae*	KX227790
B10, B26, D187	99.1–99.6	*Rickettsia *sp.	KX227788, KT032137
B12, B17, B20, B24, B218	99.6–99.8	*R. parkeri,* *R. sibrica*	KY113111, CP003341, KY124259, KY113111, HM050273
B13, B14, B15, B16, B22, D183, D186, D197, D200	96.7	*R. conorii*	AF123726
B24, D213, D214, D215, D191, D216, D127	97.6–99.1	*R. slovaca*	JX683122, KJ663756, HQ232242, CP002428, AF123723
B240, D211	99.0	*Rickettsia *sp.	KT032141, KT032136, KX227791
			
D219	99.1	*R. honei,* *R. rickettsi* *R. rhipicephali*	AF123724, CP006010, AF123719
D221	99.4	*Candidatus* *R. barbariae,* *R. slovaca,* *R. sibrica*	KY233284, AF123722
D22	98.9–99.3	*R. peacockii* *R. raoultii* *R. philipii* *R. rhipicephali*	CP001227, HQ232277, CP003308, CP013133

*Only sequences that show 96.0%–100.0% similarities with other *omp*B reference strains are reported on the above table.

### Phylogenetic analysis of rickettsial pathogens using ompB gene

**Figure 4.**
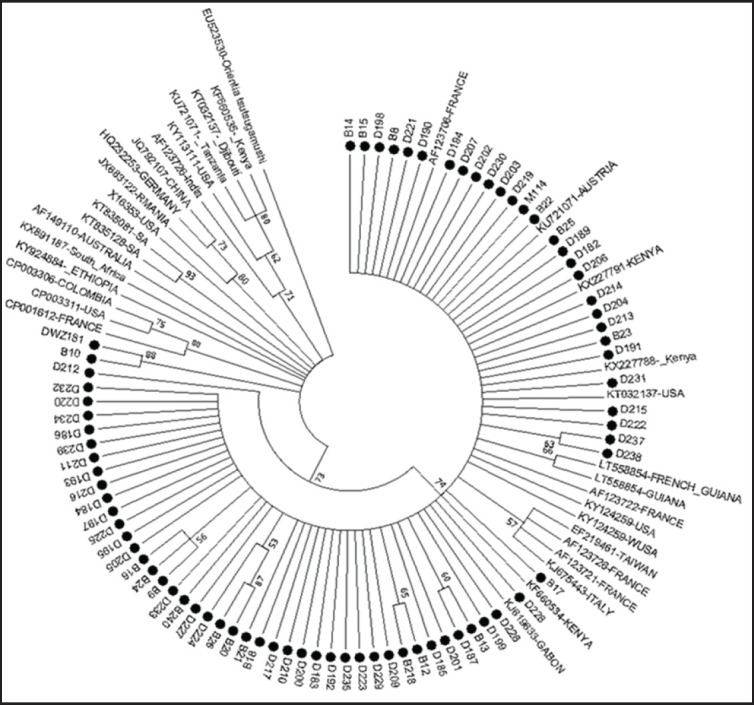
Genetic relatedness of different *Rickettsia* spp. based on the nucleotide sequence of *omp*B gene. Sequences obtained in this study are labeled with circles.

Phylogenetic analysis showed that the obtained sequences clustered into different clades with each other and with reference sequences from different geographical regions of the world. 21 sequences clustered in one clade, while another three sequences clustered with each other in a distinct clade. Sequence B9 clustered equidistance between *R. africae* [AF123706] and *R. africae* [KU721071]. Sequence B14 clustered with *Candidatus Rickettsia wissemanii* [LT558854] while D226 was found to cluster closely with *Rickettsia *sp. [EF219461]. Sequence D212 clustered with *R. raoultii* [JQ792107] while sequence D183 clustered closely between *Rickettsia* sp. [KT835128 and KT835081] with 98% bootstrap value. Finally, sequence D225 clustered with *R. rickettsii* (Fig. 4). The evolutionary history was inferred using the Maximum Likelihood method based on the Tamura-Nei model. The bootstrap consensus tree inferred from 1,000 replicates is taken to represent the evolutionary history of the taxa analyzed. Initial tree(s) for the heuristic search were obtained automatically by applying BioNJ algorithms to a matrix of pairwise distances estimated using the MCL approach and then selecting the topology with a superior log likelihood value. Evolutionary analyses were conducted in MEGA7.

The nucleotides sequences generated from this study were submitted to GenBank under the following accession numbers; MK347112 - MK347185, for *Rickettsia,* while eight representative sequences were deposited for *A. hebraeum, R. microplus, B. annulata, *and* H. longicornis* under accession number MK347205—MK347212.

## Discussion

The majority of people living in rural settlements of South Africa are in close proximity with tick-infested ruminants; hence, they are at high risk of being infected with arthropod-borne zoonotic pathogens. Cases of arthropod-borne diseases are commonly reported, especially in international travelers returning from South Africa. Therefore, it is expedient to be aware of new vectors, hosts, and pathogens [16,17].

Hard species of ticks collected in this study belonged to three genera; *Rhipicephalus, Amblyomma,* and *Haemaphysalis *were identified, with *Rhipicephalus *having the highest occurrence of 853 (71.1%), followed by *Amblyomma*; 335 (27.9%), and *Haemaphysalis*; 12 (1%). The three genera had previously been reported from South Africa [15–19]; thus, our result confirms that these genera are the predominant tick species in the study areas of studied ruminants.

Globally, various species of *Amblyomma *have been reported to be vectors of both animal and human rickettsial pathogens, which have resulted in an increased risk of SFG rickettsiosis of late [19,20]. As a rare acute and multisystemic febrile disease, spotted fever has been described to have a mortality rate of over 50% in the absence of proper prophylaxis. *A. hebraeum *with significant aggression for biting humans has been documented as a well-known vector of *R. africae*. This zoonotic tick-borne bacterial pathogen is the etiologic agent of African tick-bite fever (ATBF) in sub-Sahara Africa with a morbidity rate of >50%. Thus, after malaria,* R. africae *infection has been described to be liable for most febrile illnesses diagnosed in tourists returning from Southern Africa [11,21].

The most predominant tick species in this study was *A. hebraeum* and it had been formerly reported to be among the prevalent arthropod vectors parasitizing different animals in South Africa, especially in the Eastern Cape [18]*, *followed by* Rhipicephalus *species. The findings of Yawa et al. [18] corroborate the detection of *R. africae genetic* material in *A. hebraeum* and *Rhipicephali* species in this study, thus confirming the probable role of *A. hebraeum* and *Rhipicephali* spp. in the epidemiology of SFG Rickettsia.

The findings from the study showed that domesticated animals are in proximity to humans. They graze freely in the vegetation. Thereby, they increase the possibility of zoonosis from infected ticks to humans. However, *B. burgdorferi* sensu lato spirochetes which were profiled in the study, were not detected in the ticks’ samples analyzed. A probable reason is that the organism which is the etiologic agent of borreliosis is not present in South Africa. Though the tick vector of the pathogen has not been previously reported in South Africa, we were of the assumption that the possibility of finding it in areas close to game reserves could be high; hence we screened for it. Also, human encroachment to the wild is being reported in South Africa due to the increase in population coupled with the recent increase in animal trade and migration, hence, the possibility of this pathogen in the wild cannot be ruled out.

Furthermore, other pathogenic *Rickettsia* species (*R. africae, R. parkeri, R. mongolotimonae, R. conorii, R. honei, R. rickettsii, R. raoultii, R. australis,* and *R. rhipicephali*) similar to the SFG *Rickettsiae* were detected in different tick samples collected in this study. *R. africae* had been previously reported from ticks removed from different animals and humans in South Africa, thus its detection was expected [20,22], from its well-known vector; *A. hebraeum*. Subsequently, about 24 sequences, from the 74 obtained sequences for *omp*B gene of *Rickettsia* sp., showed between 98.9% and 99.3% homology with *R. parkeri* (KY124259, CP003341, AF123717, and KY1131111).

*Rickettsia africae*, a causative agent of ATBF, belonging to SFG *Rickettsia*, has been described as an emerging infectious pathogen in the African continent, affecting both humans and animals with devastating effects on livestock production and human health. Several seroepidemiological studies across the continent have described residence in livestock production areas as the major risk factor for seropositivity in rickettsiosis antigen [20]. The risk has been attributed to the abundance of *Amblyomma* sp. in most African countries [23], as species of *Amblyomma* have been implicated as vectors of *R. africae* infection. The increase in the percentage of infected ticks could increase the probability of humans being bitten, leading to an increased rate of human rickettsiosis.

ATBF has also been detected from American travelers’ returning from Southern Africa with a history of tick bite during their visit, as well as from positive human serum samples in western Africa [24]. The detection of *R. africae* in the present study is supported by the findings of Iweriebor et al. [20], who reported a high detection rate of *R. africae* from species of *Amblyomma *ticks collected from domesticated animals. In addition, the first detection of *R. africae, *the most widespread spotted fever agent in sub-Saharan Africa has recently been described in Corsica, France [25], through PCR from ticks that were manually removed from cattle. In the same way, Pillay et al. [26] equally reported a high incidence of *R. africae* from *Amblyomma *ticks, which has directly led to human rickettsiosis among pregnant women, although at a low incidence rate.

Similarly, the detection of *R. africae* by PCR on a skin biopsy of a returning 40-year-old Italian physician from Zimbabwe, who presented with fever and a neurological syndrome characterized by severe pain of the left leg was reported by Zammarchi et al. [27]. The global incidence rate of human rickettsiosis caused by *R. africae* has been reported to be above 5% among travelers who developed an acute febrile infection after their return from sub-Saharan Africa [24]. The presence of *R. africae *from *A. hebraeum* and species of *Haemaphysalis* and *Rhipicephalus *has long been established in South Africa [20,23,28]; hence South Africa has been described as an endemic region for ATBF.

*Rickettsia parkeri, *the causative agent of spotted fever rickettsiosis in human, was first discovered to parasitize *A. maculatum *ticks in the United States in 2004 [29], with infection in humans having similar clinical symptoms with *R. rickettsii*. Infection of humans living in the Gulf Coast (a tick endemic region) USA is very high as clinical specimen of twelve patients living in the endemic region that were submitted for laboratory evaluation confirmed six samples positive for *R. parkeri, *the etiologic agent of spotted fever rickettsiosis [30].

*R. parkeri *has also been described as a causative agent of human rickettsiosis in other countries like Colombia [31], and Brazil [32] with* Amblyomma triste* ticks haven been implicated as vectors for this infection. *R. parkeri* has equally been described as an emerging zoonotic pathogen in Mexico [33]. Similarly, Faccini-Martínez et al. [34] reported a case of *R. parkeri* infection from a Spanish traveler returning from Uruguay, who was confirmed bitten previously by *Amblyomma triste* tick. Infection by this pathogen has also been reported in Canada; hence, it has been described to be the second most important cause of tick-borne rickettsiosis in the United States, Argentina, and Brazil, after *R. rickettsia *[35]. Until now, *R. parkeri* has not been reported to infect humans on the African continent. This is likely the first study to report genetic material similar to *R. parkeri*, although from tick samples. However, with the zoonotic potential of *R. parkeri *described on other continents, it is expedient that the public be aware of its existence and appropriate authority expedite action in preventing its outbreak.

Another SFG pathogenic *Rickettsia* detected in this study was *R. mongolotimonae,* which was first recovered from *Hyalomma asiaticum* from France in 1991 [36] and in 1996. Its pathogenicity in humans was first described in a female patient with an atypical tick-transmitted disease followed by another human case from a 49-year-old HIV patient in 1998 [37].

In addition, human cases of *R. mongolotimonae* have equally been reported in Sri Lanka from a 30-year-old female who returned from traveling to a jungle and was examined as an outpatient for fever [38] and also in Cameroon from a 54-year-old woman who presented clinical symptoms of fever, headache, chills, myalgia, and arthralgia [39].

The first human case of *R. mongolotimonae* has been reported in South Africa, from a 34-year-old patient who developed a severe headache and high fever after discovering a lesion on his right foot. This rickettsiosis was linked to a bite from *H. truncatum with high* endemicity in the region where the patient had been working and is known to parasitize humans [38]. Other *Rickettsia *species associated with human diseases that have been described in South Africa include *R. conorii* and *R. sibirica*, which are etiologic agents of Mediterranean spotted fever, and North Asian tick typhus or Siberian tick typhus, respectively [20]. Despite the emergence and re-emergence of various species of *Rickettsia* with potential zoonosis, rickettsiosis is still considered a neglected disease [11].

A study conducted in Kenya by Kimita et al. [40], reported that a partial fragment of *omp*B gene was found to be the most identical to *Rickettsia rhipicephalus* with 99.0% homology as against a required homology of 99.2% to qualify it as *R*. *rhipicephalus,* thus suggesting the probability of *R*. *rhipicephalus *circulating in the African continent. *Rhipicephalus* spp. has been described as the main arthropod vector for this bacterium in different geographical regions, which could probably be distributed by migratory birds and wild animals. Similarly, human infections with *R. conorii* have been described in some European countries such as France, Spain, Portugal, and Greece [41]. The detection of *R. conorii* from French athletes, who returned from South Africa and presented with headache, fever, regional lymphadenopathies, and multiple inoculation eschars was also reported [38].

The detection of *R. conorii* in different *Rhipicephalus* spp. in the study areas implies a wide range of its host and ecological variation which does have epidemiological consequences. Also, the populace living in proximity to domesticated animals in the study area is at high risk of rickettsial infection if bitten by infected ticks, as the presence of genetic materials of the organisms detected in this study indicates probable zoonotic potential. Therefore, a systematic study is further required to establish the detection of these pathogens from human samples.

Several studies on ticks have been previously conducted in South Africa, which has shown that varieties of SFG are in circulation in the country [15,20,42]. Similarly, a recent study conducted by Essbauer et al. [11] showed that different species of pathogenic *Rickettsia* spp. are in circulation in the country.

## Conclusion

This study revealed the diversity of *Rickettsia* spp. and the presence of *R. parkeri*, *R. australis*, and *R. mongolotimonae*; all belonging to the SFG Rickettsia for the first time in the Eastern Cape Province, South Africa, thus suggesting a potential role for *A. hebraeum* and *Riphicephalis* spp. as vectors in the area. Owing to the increase in demand for livestock in international trades, systematic surveillance is highly recommended for the update of epidemiological data of these emerging and re-emerging arthropod-borne pathogens. The limitation of the study is the amplification and analysis of only *omp*B gene, and this is due to the limited availability of financial resources.

## List of abbreviations

ATBF, African tick-bite fever; DNA, Deoxyribonucleic acid; MCL, Maximum composite likelihood; RNA, Ribonucleic acid; SFG, Spotted fever group; TBD, Tick-borne diseases; TBPs, Tick-borne pathogens; TG, Typhus group.
